# Antiviral Trials—Navigating the Shifting Sands of a Pandemic

**DOI:** 10.1093/cid/ciaf031

**Published:** 2025-02-17

**Authors:** Beatrice Z Sim, Cameron R Wolfe

**Affiliations:** Division of Infectious Diseases, Department of Medicine, Duke University, Durham, North Carolina, USA; Sir Peter MacCallum Department of Oncology, University of Melbourne, Parkville, Victoria, Australia; National Centre for Infections in Cancer, Melbourne, Victoria, Australia; Division of Infectious Diseases, Department of Medicine, Duke University, Durham, North Carolina, USA

**Keywords:** COVID-19, ensitrelvir, antivirals

**(See the Major Article by Luetkemeyer et al. on pages 1235-44.)**

The coronavirus disease 2019 (COVID-19) pandemic tested the resiliency and traditionally versatile infectious diseases community, both in our management of the public health response and in how we adapted as a research community to the urgency, magnitude, and visibility of the problem. While we will deservedly look back on many successes, the COVID-19 landscape has changed significantly since the beginning of the pandemic. As we look ahead to development of additional countermeasures for severe acute respiratory syndrome coronavirus 2 (SARS-CoV-2) and think about future emergent threats, we must also adapt how we develop our clinical trial pipeline.

In this edition of *Clinical Infectious Diseases*, Luetkemeyer et al [1] report the results of the SCORPIO-HR phase 3 trial, in which the efficacy and safety of ensitrelvir, a SARS-CoV-2 protease inhibitor, was evaluated in a population of nonhospitalized symptomatic adults with and without risk factors for progression to severe disease. This drug is functionally similar to other approved protease inhibitors, for example, nirmatrelvir and simnotrelvir, and was approved in Japan on the basis of the SCORPIO-SR trial [2].

The authors found that, despite demonstration of virological benefit (lower viral loads, proportionally more negative viral cultures in treated patients compared with placebo), there was no statistically significant reduction in the primary end point, that is, time to sustained symptom resolution on a 15-point scale. This scale, developed during earlier COVID-19 trials, includes symptoms that range from rhinorrhea to dyspnea (Table 1). Reassuringly, rates of adverse events remained low in both groups, consistent with prior trials of ensitrelvir and other protease inhibitors. In short, the trial by Luetkemeyer et al suggests that at this point in the pandemic, with so many previously infected or vaccinated patients enrolled, ensitrelvir may not be helpful in this mixed-risk patient population. However, the drug appears safe, and ideally, we believe it should remain in our armamentarium in the event of another similar pandemic.

During this later arc of the pandemic, numerous well-conducted, multinational, randomized, placebo-controlled trials have also failed to reach their primary end points. Nirmatrelvir/ritonavir, which showed positive efficacy in a largely unvaccinated outpatient population early in the pandemic, failed to meet similar primary end points (time to sustained alleviation of COVID-19 signs and symptoms) in a phase 2–3 study of standard-risk patients [3]. Bemnifosbuvir, a nucleotide analog, did not meet its time-to-symptom-alleviation/improvement primary end point, though it demonstrated promise in its phase 2 study, also conducted at an earlier pandemic time point [4, 5].

The difficulty of adapting to the rapidly changing population and virus likely contributes to ultimate trial failure. The most significant population change was community vaccination, which saw hospitalization and mortality fall. With effective global vaccination strategies and evolving natural immunity, well-designed trials may fail when their biologically plausible end points (such as intensity and duration of symptoms), designed based on earlier phases of the pandemic, are no longer relevant by the end of the trial. SCORPIO-HR was conducted when the Omicron variant was dominant and there was a 76% rate of vaccination, potentially accounting for some of the scant clinical benefit seen on the 15-point symptom scale, which was designed based on earlier trial results with different variants and different trial participant immunity. While these symptoms were a priority early in the pandemic, our research community was potentially slow to adapt trial end points, not only to mirror evolving symptoms and risks but to ultimately reflect end points most relevant to contemporaneous patients.

Critically, what is defined as a “high-risk population” not only evolved during the COVID-19 pandemic but will likely do so over the course of any future pandemics, especially with efforts to deploy effective vaccines. The most common risk factors for progression to severe COVID-19 in the EPIC/HR trial for nirmatrelvir/ritonavir were body mass index ≥ 25 kg/m^2^, current smoking status, and hypertension [6]. The risk factors in the PINETREE trial for remdesivir were diabetes mellitus, obesity, and hypertension [7]. Conversely, for SCORPIO-HR, outside the United States, obesity was the most common risk factor for progression. When the populations in the keystone trials that inform our antiviral strategies simply no longer mirror those in the current phase of the pandemic, we must adapt both trial design and patient recruitment accordingly.

The SARS-CoV-2 virus also changed over the course of the pandemic, likely further dynamically impacting end points as the SCORPIO-HR trial progressed. Luetkemeyer and colleagues should be commended for trying to track virologic end points. However, the relevance of virological measurements in the absence of concomitant clinical change remains unclear, and these findings do not seem to meaningfully impact regulatory deliberations, at least at the present time.

All of this begs the question: how does one navigate the shifting sands of a pandemic, run a clinical trial, fulfill the requirements of multiple regulatory authorities, and gather evidence for potential use and approval of a drug when the viral and population targets are continually changing over the arc of the trial? Traditional trial formats and established regulatory approval pathways have not proven nimble. The establishment and maintenance of multicenter and likely multinational trial networks will be vital for rapid collection and pooling of data. Standardized definitions to aid easy comparison of trials are essential.

However, the largest unmet opportunity is likely for trial teams and regulatory authorities to be creative in building adaptive design into protocols that cannot only move with the evolution of therapeutics but also with the clinical landscape. This will be vital for the next pandemic. For example, adaptive trial design is not new to infectious diseases, but adaptive end point selection is not routinely recommended by regulatory bodies due to the risk of bias [8]. Population enrichment is more commonly used but could be incorporated more frequently into these antiviral clinical trials [9]. Modified regulatory opportunities that are capable of handling modified trial design should be explored in preparation for future pandemics as we reflect on our successes and trial failures of the last few years.

It is important not to underestimate the work done by Luetkemeyer et al. This trial, despite failing to achieve a stated primary end point, not only corroborates safety data for ensitrelvir but highlights many of the challenges experienced by clinician researchers and pharmaceutical companies as the pandemic evolved. Importantly, we hope that a negative trial does not portend the demise of a drug that may hold important value in a future coronavirus pandemic or transmission wave. In an optimal population with an alternate epidemiological landscape and with contemporaneous end points, it may prove beneficial. SCORPIO-HR hopefully can also catalyze ongoing federal discussion surrounding deft emergent trial design, nimble regulatory pathways, and patient-centered outcomes.

## Note

***Financial support***. B. Z. S. is supported by a National Health and Medical Research Council post-graduate scholarship (2031043).
